# Prevalence of Thymine—Adenine Dinucleotide Repeat, IL28B and IFNL4 in Thai Population and Correlation with Spontaneous Clearance and Treatment Outcome of Hepatitis C Infection

**DOI:** 10.1371/journal.pone.0125400

**Published:** 2015-05-04

**Authors:** Vo Duy Thong, Rujipat Wasitthankasem, Pisit Tangkijvanich, Sompong Vongpunsawad, Yong Poovorawan

**Affiliations:** 1 Center of Excellence in Clinical Virology, Faculty of Medicine, Chulalongkorn University, Bangkok, Thailand; 2 Research Unit of Hepatitis and Liver Cancer, Department of Biochemistry, Faculty of Medicine, Chulalongkorn University, Bangkok, Thailand; 3 Department of Internal Medicine, Division of Gastroenterology and Hepatology, Faculty of Medicine, University of Medicine and Pharmacy, Ho Chi Minh, Vietnam; University of Sydney, AUSTRALIA

## Abstract

**Background:**

The interleukin-28B (*IL28B*) gene polymorphism is a strong baseline predictor of sustained virological response (SVR) in hepatitis C virus (HCV) treatment. The length of thymine—adenine dinucleotide repeats, or (TA)_n_, in the regulatory region of *IL28B* can affect interferon transcription. In order to determine predictive values in HCV infection, we explored the correlation among factors including (TA)_n_ genotypes, clinical features, interferon-λ-3 (IFNL3) and interferon-λ-4 (IFNL4) polymorphisms, and HCV treatment outcome.

**Methods:**

Sera from 492 patients with chronic HCV infection, 101 individuals with spontaneous HCV clearance and 123 healthy blood donors (control group) were analyzed. Genotyping of the (TA)_n_ was performed by direct sequencing. The rs12979860 (IFNL3) was identified using nested PCR and sequencing, while ss469415590 (IFNL4) was identified by real-time PCR.

**Results:**

The distribution of (TA)_n_ was similar between individuals with spontaneous HCV clearance and chronic HCV infection, but differed significantly from healthy controls. Individuals with both (TA)_n_ alleles ≥12 had significantly higher SVR rate compared to individuals with at least one (TA)_n_ <12 allele. This strong correlation was seen for patients infected with HCV-1, HCV-3, and HCV-6. The (TA)_n_ genotypes were not associated with HCV viral load, ALT levels and liver stiffness, but were correlated with platelet counts (*p<0*.*001*). In contrast, rs12979860 (CC) and ss469415590 (TT/TT) genotypes were associated with higher SVR rated only in patients with HCV-1.

**Conclusions:**

The (TA)_n_ genotypes were not associated with spontaneous clearance of HCV infection but associated with treatment response in patients infected with HCV-1, HCV-3 and HCV-6. In contrast, IFNL3 and IFNL4 polymorphisms were predictive of treatment outcome only for patients infected with HCV-1.

## Introduction

Hepatitis C virus (HCV) infection is a significant global public health problem affecting an estimated 160 million people (~2.35% of the population) worldwide [1]. The progression to chronic HCV sometimes necessitates the need for liver transplantation and is often the leading cause of hepatocellular cancer [2]. A combination of pegylated interferon (PEG-IFN) combined with ribavirin (RBV) for the duration of 24 to 48 weeks has been the standard-of-care therapy for HCV infection for the past decade. Virus genotype and host factors such as age, sex, race, fibrosis, and steatosis can determine the treatment outcome [3–5]. In 2009, several genome-wide association studies reported that single nucleotide polymorphisms (SNPs) upstream of the interleukin-28B (*IL28B*) gene, which encodes interferon-λ-3 (IFNL3), were strongly associated with response to PEG-IFN/RBV therapy and spontaneous HCV clearance [6–8]. The rs12979860 of CC genotype is associated with a two-fold greater sustained virological response (SVR) rate than the TT genotype [6]. Interestingly, the gene frequency of C allele is much higher in European and Asian ancestries than in African ancestry. Recently, it was shown that the polymorphism in IFN-λ-4 (IFNL4) gene, ss469415590 of TT genotype, is more strongly associated with treatment-induced response and spontaenous HCV clearance than rs12979860 in Europeans and Asian, but especially in individuals of African ancestry [9].

Recently, an insertion/deletion polymorphism in the promoter region of *IL28B* consisting of thymine—adenine dinucleotide repeats (TA)_n_ has been linked to *IL28B* gene expression. The length of (TA)_n_ reportedly varies from 10 to 18 repeats with the most frequent genotype of 12/12 [10]. Luciferase assay showed that the transcriptional activity of the promoter increased gradually with increasing (TA)_n_ length. Therefore, (TA)_n_ could be associated with the transcriptional activity of *IL-28B* and could potentially be used to improve predictions of the response to interferon-based HCV treatment. In this study, we focused on the distribution of the length of (TA)_n_ and the correlation of (TA)_n_ genotypes with treatment outcome, clinical features, *IL28B* and IFNL4 polymorphisms in HCV infection.

## Patients and Methods

### Patients

**Fig 1:**
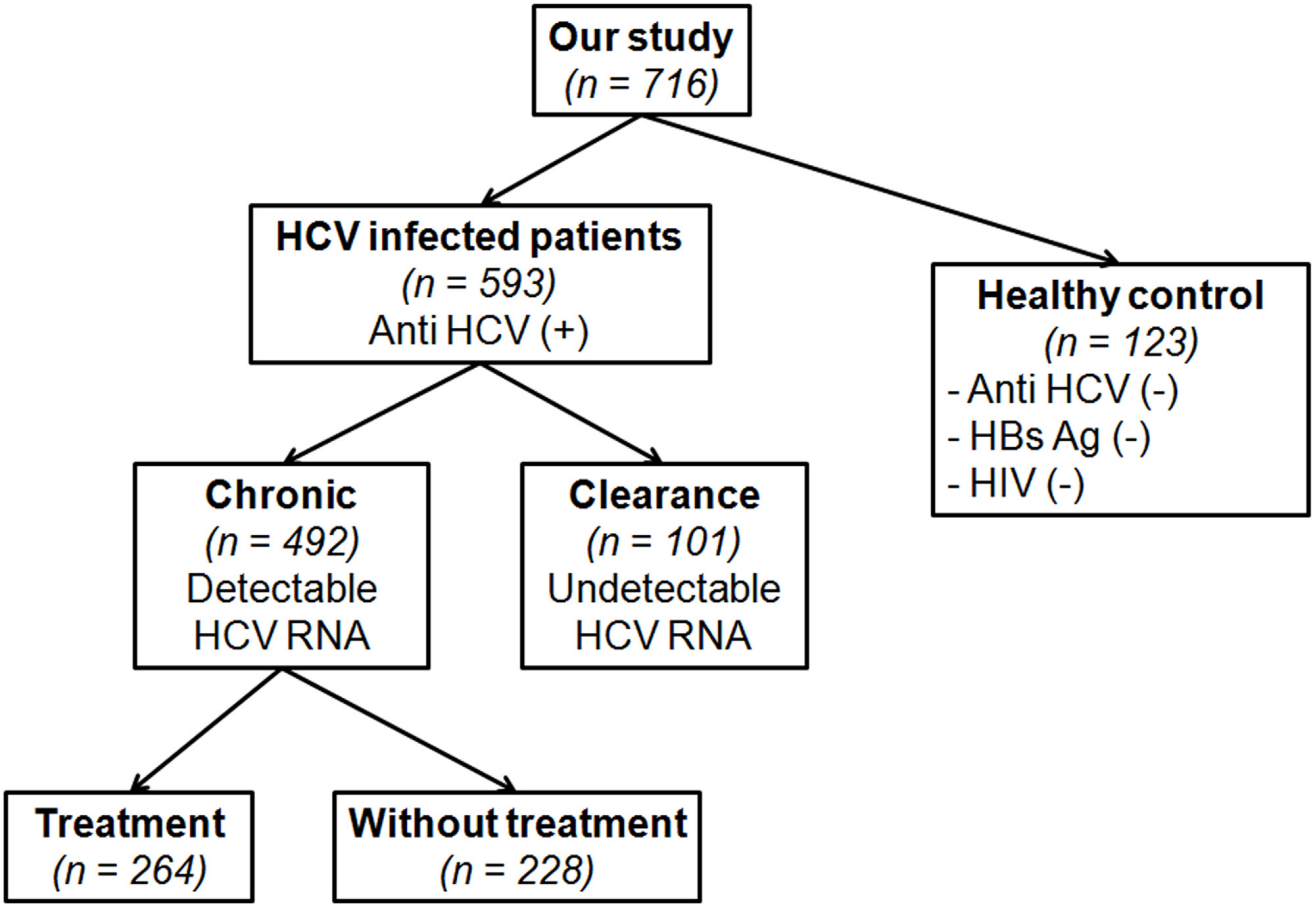
Diagram on the collection of cohort in this study.

The study followed the Helsinki Declaration on medical research. The study obtained written informed consents from patients and the protocol was approved by the Institutional Review Board of the Faculty of Medicine, Chulalongkorn University (IRB No. 517/57). A total of 593 HCV-infected Thai individuals comprising of 101 patients with spontaneous HCV clearance (defined as anti-HCV sero-positive and undetectable HCV RNA in patients without previous antiviral treatment) and 492 patients with chronic HCV (defined as anti-HCV sero-positive and detectable HCV RNA for more than 6 months) were followed-up at King Chulalongkorn Memorial Hospital in Bangkok, Thailand. Among the 492 patients chronically infected with HCV, 264 underwent treatment and 228 did not. Patients who received ≥ 80% of the recommended dose of PEG-IFN/RBV were considered assessable for response to treatment. SVR was defined as an absence in detectable HCV RNA in serum at 24 weeks after treatment termination. All other patients were considered non-responders. For comparison, 123 healthy Thai blood donors who tested negative for HBsAg and anti-HCV comprised the control group (Fig 1). Among these, 225 individuals from a previous study to investigate the association of rs12979860 and ss469415590 with HCV treatment response [11] were included in this study for better data analysis.

### Methods

#### Clinical, biochemical, liver assessment evaluation and SNP genotyping

During screening, we obtained patient information including demographical data (age, sex, height, weight, and body mass index) and laboratory tests at baseline including blood cell counts, aspartate transaminase (AST) and alanine transaminase (ALT). Liver fibrosis was evaluated through fibroscan stiffness. HCV treatment was also extracted from clinical database.

Two SNPs, rs12979860 (*IL28B*) and ss469415590 (IFNL4), were genotyped using nested PCR [11] and real-time PCR [9], respectively. For rs12979860, PCR products were sequenced and the major (CC) and minor (TT) alleles were determined. In order to investigate an upstream variation of *IL28B*, ss469415590, real-time PCR using the Taqman genotyping assay with MGB probes (Applied Biosystems, Carlsbad, CA) was performed as previously described [9, 12]. TT and ΔG were major and minor alleles, respectively.

#### HCV RNA quantification and genotypes

HCV RNA quantification was performed using the real-time quantitative reverse-transcription polymerase chain reaction (qRT-PCR) (COBAS TaqMan HCV assay, Roche Diagnostics, Basel, Switzerland) according to the manufacturer’s instructions.

HCV genotypes were determined by nucleotide sequencing of the core and NS5B regions followed by phylogenetic analysis as previously described [13].

#### TA repeat genotyping

Genomic DNA of patients was extracted from peripheral blood mononuclear cells (PBMC) or plasma using the QIAamp DNA Mini Kit (Qiagen, Hilden, Germany). PCR was performed using primers TA-CU-R (5’-CAATTCTTGAGCAGAGCCTCA-3’) and TA-CU-F (5’- GGAAGGTATGTTCCCAAGAGG-3’) and contained 5 μL DNA, 5 pmol of each primer, 10 μl of 2x Perfect *Taq* Plus MasterMix (5 PRIME, Gaithersburg, MD) in a total volume of 25 μl. The amplification cycles were: 94°C for 5 min, followed by 40 cycles of denaturation at 94°C for 30 sec, annealing at 54.9°C for 30 sec, extension at 72°C for 30 sec, and a final extension at 72°C for 7 min. PCR fragment was resolved by 2% agarose gel electrophoresis. PCR products were purified using GelExtract Mini Kits (5 PRIME, Gaithersburg, MD) and subjected to sequencing (First BASE Laboratories, Selangor, Malaysia) using both forward and reverse primers. The length and genotypes of TA repeat were analyzed manually based on the chromatograms (Chromas LITE, version 2.01) and compared to the reference sequence retrieved from GenBank (http://www.ncbi.nlm.nih.gov/).

#### Data analysis

The Mann-Whitney U test or Student’s test was used to compare continuous variables, and the χ2 test or Fisher’s exact test was used to compare categorical variables. The Spearman rank correlation was used to evaluate the relationships among variables. All data were analyzed using SPSS Statistic Software Package for Windows version 20.0 (SPSS, Chicago, IL). Statistical significance was set at *p<0*.*05* and all tests were two-tailed. Numerical data are presented as mean +/- standard deviation (SD) or median and interquartile range (IQR).

## Results

Patients with spontaneous HCV clearance, chronic HCV, and the control group had comparable mean age, body mass index (BMI), and *IL28B* genotype distribution (Table 1). Chronic HCV group had slightly different IFNL4 and (TA)_n_ genotype distribution compared to other groups, but these values were not statistically significant. However, twice as many males than females had chronic HCV (*p<0*.*001*).

**Table 1:** Demographics and characteristics of healthy controls, patients with spontaneous HCV clearance, and patients with chronic HCV.

Characteristic		All (n = 716)	Control (n = 123)	Spontaneous Clearance (n = 101)	Chronic HCV (n = 492)	*p value*
**Age, years**		44.78 ± 10.1	46.76 ± 5.8	41.17 ± 10.9	44.89 ± 10.7	0.351
**Sex**						0.015
	Males	460 (64.2%)	73 (59.3%)	53 (52.5%)	334 (67.9%)	
	Females	256 (35.8%)	50 (40.7%)	48 (47.5%)	158 (32.1%)	
**BMI, kg/m** ^**2**^		25.33 ± 11.2	N/A	24.13 ± 4.2	25.59 ± 12.2	0.327
**rs12979860 genotypes**						NS
	CC	624 (87.2%)	109 (88.6%)	88 (87.1%)	427 (86.8%)	
	CT	76 (10.6%)	13 (10.6%)	11 (10.9%)	52 (10.6%)	
	TT	16 (2.2%)	1 (0.8%)	2 (2%)	13 (2.6%)	
**ss469415590 genotypes**						NS
	TT/TT	615 (85.9%)	110 (89.4%)	90 (89.1%)	415 (84.3%)	
	TT/ΔG	86 (12.0%)	12 (9.8%)	9 (8.9%)	65 (13.2%)	
	ΔG/ΔG	15 (2.1%)	1 (0.8%)	2 (2%)	12 (2.4%)	
**(TA)**_**n**_ **genotypes**						NS
	Hetero-/homozygous	37/679 (5.2%/94.8%)	12/111 (9.8%/90.2%)	9/98 (8.9%/91.1%)	16/476 (3.3%/96.7%)	

In parentheses are percentages unless otherwise noted. p-value <0.05 is considered statistically significant. N/A = Information not available. NS = not significant.

Homozygous is defined as having two identical alleles.

Heterozygous is defined as having different alleles.

### Overall distribution of the allele frequencies of (TA)_n_ in the study population

**Fig 2:**
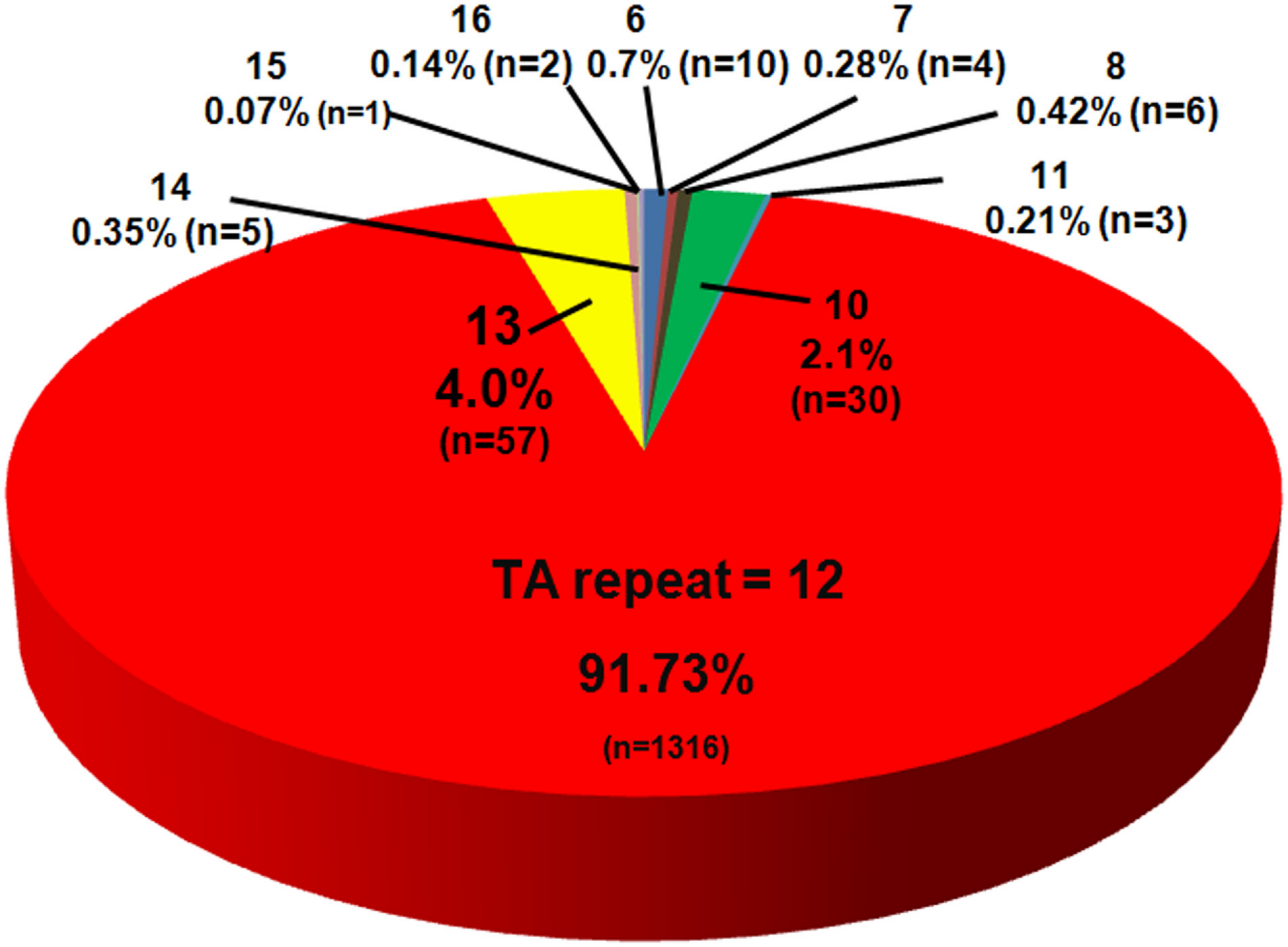
Allele frequencies of (TA)_n_ in this study (n = 1432).

When both alleles from all 716 individuals in this study were examined (1432 alleles altogether), the observed variation of (TA)_n_ ranged from 6 to 16 with the mode of 12 (91.7%) (Fig 2). The second and third most frequent (TA)_n_ were 13 (4.0%) and 10 (2.1%), respectively. Other (TA)_n_ genotypes comprised less than 1% each, and no (TA)_n_ of 9 was observed.

**Fig 3:**
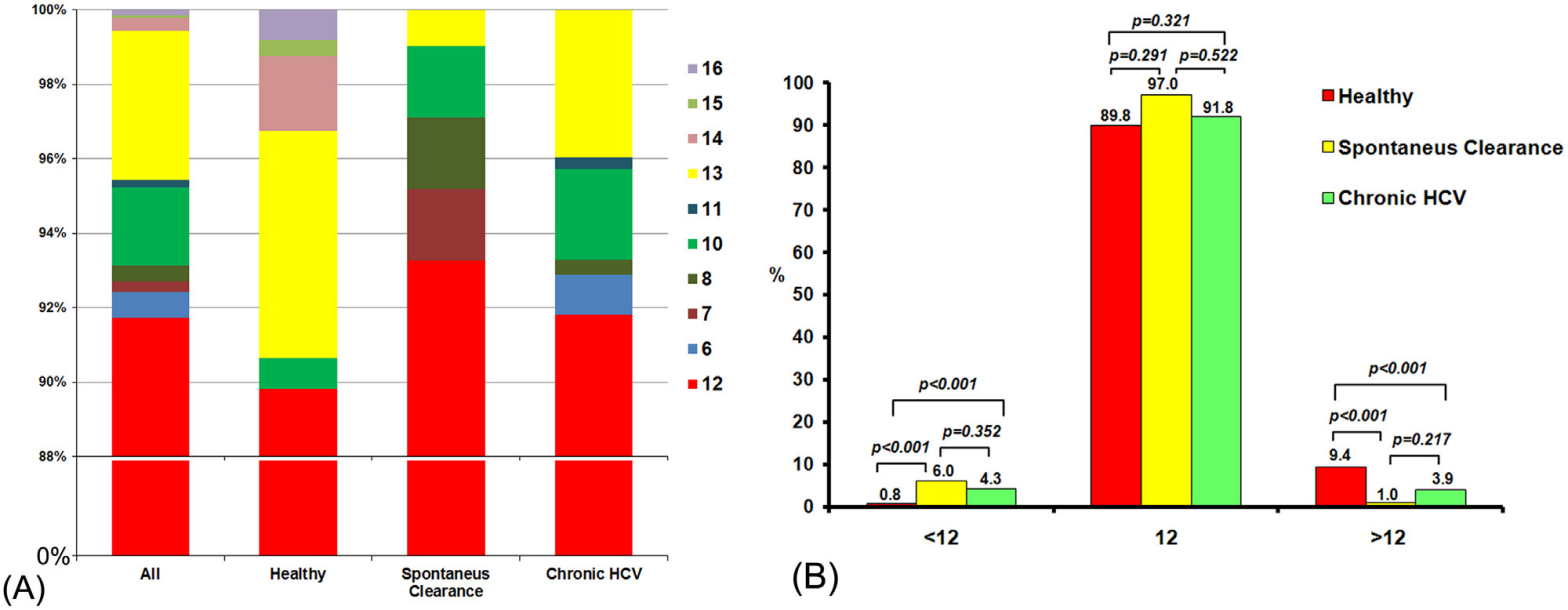
Allele distribution in our population. (A) Allele frequency of (TA)_n_ distribution in the control, spontaneous clearance, and chronic HCV groups (n = 1432). (B) The frequency distribution of (TA)_n_ genotypes with <12, 12 and >12 alleles in control, spontaneous clearance, and chronic HCV groups (n = 1432).

Genotype (TA)_n_ of 12 was predominant in all 3 groups (Fig 3A). Although all individuals had (TA)_n_ of 13, individuals in the spontaneous clearance and chronic HCV groups did not have (TA)_n_ >13. As a result, these groups had significantly fewer (TA)_n_ >12 than the control group (*p<0*.*001*). Furthermore, (TA)_n_ <10 was not observed in the control group. Comparison between the spontaneous clearance and chronic HCV groups showed that there was no significant difference in the frequency of allele (TA)_n_ >12 (*p = 0*.*217*) or allele (TA)_n_ <10 (*p = 0*.*352*) (Fig 3B).

### Prevalence of (TA)_n_ genotypes in spontaneous clearance, chronic HCV, and control groups

For the purpose of analysis, we defined an individual as homozygous for (TA)_n_ genotype when that person possessed the same (TA)_n_ for both alleles. In contrast, an individual is heterozygous for (TA)_n_ genotype when that person possessed different (TA)_n_ alleles. When all 3 groups were analyzed, we found that the (TA)_n_ genotype was 94.8% homozygous (679/716) and 5.2% heterozygous (37/716). The most prevalent (TA)_n_ homozygous genotype was 12/12, meaning that an individual possessed (TA)_n_ of 12 for both alleles. When analyzing each group separately, there were more individuals with (TA)_n_ of 12/12 in the spontaneous clearance group than in the control or chronic HCV groups (97.0%, 89.8%, and 91.1%, respectively).

**Fig 4:**
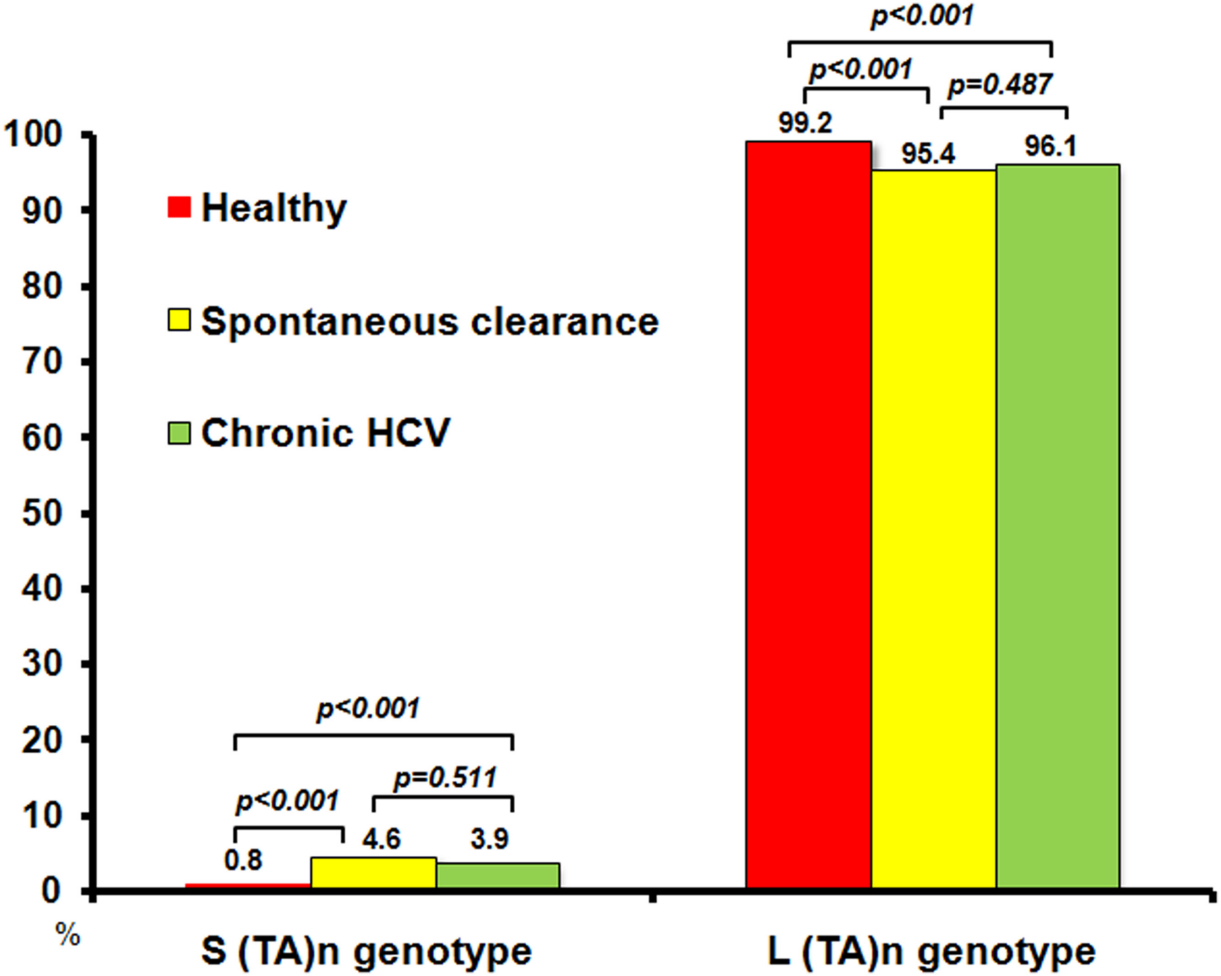
Prevalence of (TA)_n_ genotypes in control, spontaneous clearance, and chronic HCV groups (n = 716). The (TA)_n_ genotype is defined as “L” (when both alleles are ≥ 12) and “S” (when at least one allele is <12).

To further simplify the analysis for individuals with heterozygous (TA)_n_, we defined an individual genotype "L" when both alleles were ≥ 12 and “S” when at least one allele is <12. Using this definition, the most prevalent heterozygous (TA)_n_ genotype in all 3 groups was L (Fig 4). Genotype L was less frequent in the spontaneous clearance (94.4%) and chronic HCV (95.2%) groups than the control group (99.2%) (*p<0*.*001*). The difference in genotype L between spontaneous clearance and chronic HCV groups was not statistically significant (*p = 0*.*487*). In contrast, genotype S was more frequent in the spontaneous clearance (4.61%) and chronic HCV (3.85%) groups compared to the control group (0.81%) (*p<0*.*001*).

### SVR rates according to (TA)_n_, rs12979860 and ss469415590

**Fig 5:**
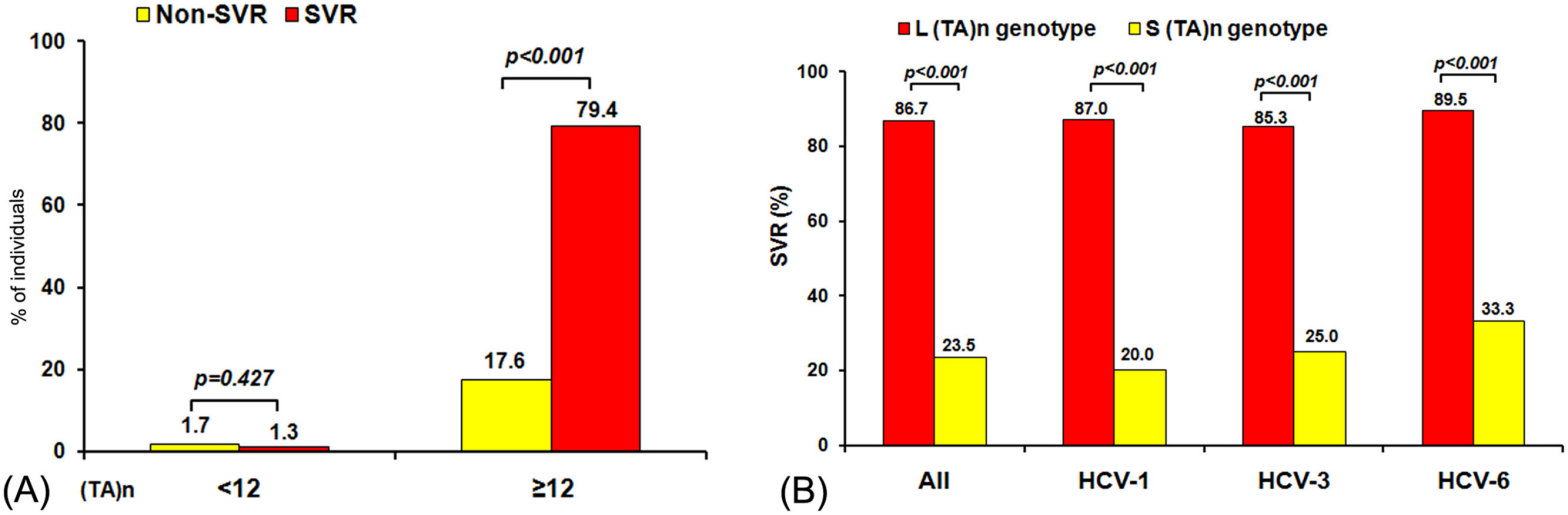
Association of (TA)_n_ with SVR. (A) Association of (TA)_n_ with SVR to PEG-IFN/RBV therapy among individuals with chronic HCV. (B) Percentage of (TA)_n_ genotypes L and S relative to SVR in individuals with chronic HCV who underwent PEG-IFN/RBV therapy. The (TA)_n_ genotype is defined as “L” (when both alleles are ≥ 12) and “S” (when at least one allele is <12).

The 264 individuals with chronic HCV underwent PEG-IFN/RBV therapy. When the (TA)_n_ <12, SVR and non-SVR rates were similar (1.70% versus 1.33%, *p = 0*.*427*) (Fig 5A). In contrast, individuals with (TA)_n_ ≥12 were more likely to have SVR than non-SVR (79.36% versus 17.61%, *p<0*.*001*). Furthermore, individuals with genotype L had significantly higher SVR rate than those with genotype S (84.9% vs. 53.8%, *p<0*.*001*). Collectively, this observation shows striking concordance for all HCV genotypes (HCV-1: 87.0% vs. 20.0%, *p<0*.*001*; HCV-3: 85.3% vs. 25.0%, *p<0*.*001*; and HCV-6: 89.5% vs. 33.3%, *p<0*.*001*) (Fig 5B).

**Fig 6:**
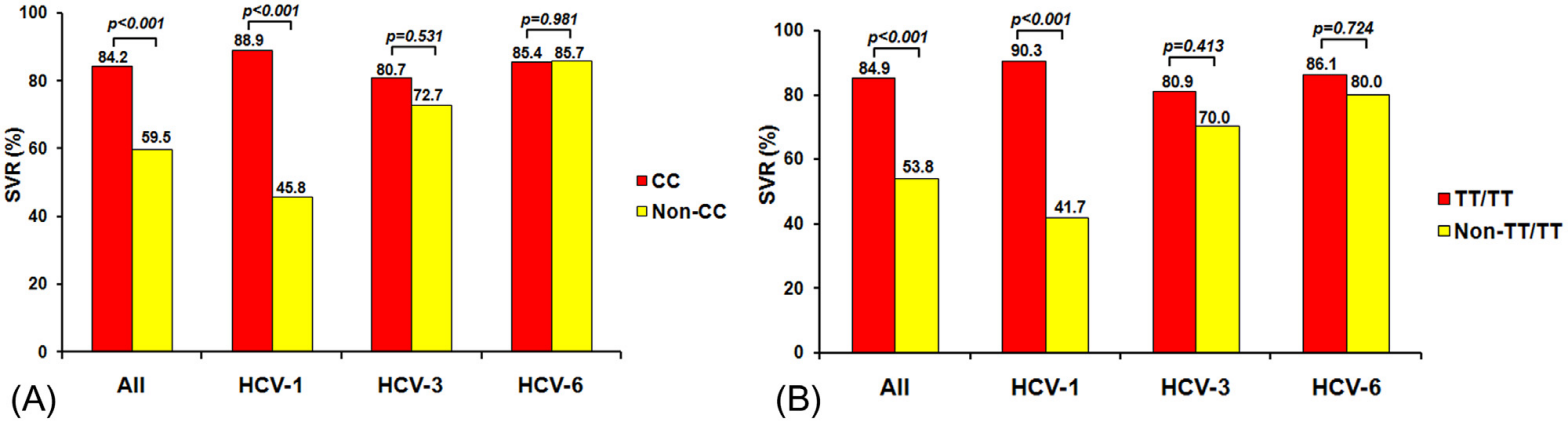
Association of rs12979860 genotype (A) and ss469415590 genotypes (B) with SVR to PEG-IFN/RBV therapy in patients with HCV infection.

Overall, significantly higher SVR rates were observed in chronic HCV with the favorable CC genotype than non-CC genotype for rs12979860 (84.2% vs. 59.5%, *p<0*.*001*) and TT/TT genotype than non-TT/TT genotype for ss469415590 (84.9% vs. 53.8%, *p<0*.*001*) (Fig 6A and 6B). The differences in SVR between favorable and unfavorable genotypes for both rs12979860 and ss469415590 were greatest for HCV-1. For rs12979860, 88.9% of patients with CC genotype achieved SVR compared to 45.8% of patients with non-CC genotype (*p<0*.*001*). For ss469415590, 90.3% of patients with TT/TT genotype achieved SVR compared to 41.7% of patients with non-TT/TT (*p<0*.*001*). The differences in SVR for HCV-3 and HCV-6, however, were not statistically significant. Although results from our previous study suggested association between rs12979860 *IL28B* and ss469415590 IFNL4 (*p<0*.*001*), these polymorphisms were not associated with (TA)_n_ (*p = 0*.*129* with rs12979860, *p = 0*.*108* with ss469415590 IFNL4, respectively).

### Association of (TA)_n_ genotypes with clinical parameters

Several clinical parameters were examined in patients with chronic HCV (Table 2). Individuals with (TA)_n_ genotypes S and L did not demonstrate statistically significant differences in hemoglobin (*p = 0*.*975*) or serum ALT levels (*p = 0*.*199*). Similarly, (TA)_n_ genotypes did not correlate with liver stiffness (*p = 0*.*294*) or HCV RNA (*p = 0*.*305*). However, (TA)_n_ genotype S correlated with increased platelet counts (*p<0*.*001*).

**Table 2:** Correlations between (TA)_n_ genotypes and clinical parameters.

Characteristic	TA repeat genotypes		*p value*
	S (TA)_n_ genotype	L (TA)_n_ genotype	
**Hemoglobin, g/dL**	14.16±1.2	14.19±1.5	0.957
**ALT, U/L**	61.27±54.2	85.70±61.6	0.199
**Platelets, × 10** ^**9**^ **per liter**	265.45±64.2	197.12±62.1	<0.001
**HCV RNA, log**_**10**_ **IU/ml**	5.60±1.3	5.88±0.9	0.305
**Liver stiffness, kPa**	7.04±4.5	9.22±5.6	0.294

p-value <0.05 are considered statistically significant

## Discussion

Host genetic factors can affect the outcome of HCV infection resulting in either spontaneous clearance from acute infection without treatment or persistence leading to chronic HCV and liver cirrhosis. Polymorphisms near the *IL28B* gene can determine the outcome of infection and the response to treatment. In the present study, we explored the overall prevalence of (TA)_n_ genotypes among Asians of Thai descent with HCV infection resulting in natural clearance or chronic HCV. We found that the variation of (TA)_n_ ranged from 6 to 16 and the most frequent (TA)_n_ was 12 (91.73%) in our population. This finding was consistent with an earlier study in a Japanese cohort, which also found that 75% of individuals examined possessed (TA)_n_ of 12 [10]. The allele <10 (TA)_n_ was significantly more frequent in the spontaneous clearance and chronic HCV groups than in the healthy controls (*p<0*.*001*). This difference may in part be attributed to the genetic background represented by the fewer number of controls (n = 123) compared to infected individuals (n = 593). Otherwise, the allele >12 (TA)_n_ was found in significantly higher number in healthy individuals group compared with spontaneous clearance and chronic HCV groups (*p<0*.*001*). Although the determination of the (TA)_n_ genotypes was performed manually from chromatograms, two independent sequencing experiments were done for each sample to ensure data reproducibility.

Previous studies found that transcription of *IL-28B* was upregulated in the CC genotype of rs12979860, which was associated with SVR [7, 8, 14], suggesting that the expression levels of *IL-28B* could be one of the key factors to clear HCV under PEG-IFN/RBV therapy and could also affect spontaneous clearance of acute HCV infection [15], whereas the length of (TA)_n_ in the regulatory region of *IL-28B* could affect the regulation of *IL-28B* transcription [10]. The most prevalent (TA)_n_ genotype in our population was when both alleles were ≥12, with higher frequency in healthy individuals (99.2%) compared to spontaneous clearance (94.4%) and chronic HCV (95.2%) (*p<0*.*001*). The distribution of the (TA)_n_ genotype S (when at least one allele was <12) was similar among HCV-infected individuals, but interestingly, the (TA)_n_ genotype S was significantly more frequent in the spontaneous clearance and chronic HCV groups than in the healthy controls (*p<0*.*001*). Although we confirmed previous observations that favorable SNPs rs12979860 (CC) and ss469415590 (TT/TT) strongly correlated with improved SVR with HCV-1, but not HCV-3, and HCV-6, we found that (TA)_n_ ≥12 correlated with increase SVR for HCV-1, -3, and -6.

A recent study from Japan demonstrated the promise of (TA)_n_ genotype in predicting spontaneous HCV clearance [16]. The most frequent allele of (TA)_n_ found in that Japanese cohort was also 12, which accounted for approximately 80% of individuals. In contrast, African-American cohort in that study demonstrated a gradient of (TA)_n_ alleles ranging from 6 to 18, and although allele 12 was the most common, it only accounted for 30% of the individuals. More importantly, African-Americans with longer (TA)_n_ were significantly associated with spontaneous HCV clearance, which attest to the promise of the predictive ability of (TA)_n_ towards desirable clinical outcome in HCV infection.

Several SNPs in linkage disequilibrium upstream or within the *IL28B* gene on chromosome 19q13 are strongly associated with SVR to PEG-IFN/RBV therapy [7, 8, 14]. One such polymorphism, rs12979860 (CC) genotype, is associated with greater rate of SVR than CT or TT genotypes in European-Americans, African-Americans, and Hispanics infected with HCV-1 [6]. In particular, African-Americans with the CC genotype responded better to treatment than European-Americans with the TT genotype, suggesting that an individual’s rs12979860 genotype is a better predictor of SVR than ethnicity. Furthermore, it is a better predictor of HCV clearance, whether natural or in response to treatment, than the baseline viral load or fibrosis. This finding has been confirmed with Egyptians, Europeans, and Sub-Sahara Africans infected with HCV-4 [17, 18].

Another variant in the upstream region of *IFNL3*, designated as *IFNL4*, is also associated with treatment efficacy in HCV-infected patients [9]. This region, ss469415590, harbors a dinucleotide variant that is found in two alternative forms (ΔG or TT alleles). The ss469415590 is more strongly associated with treatment response of patients infected with HCV-1 than rs12979860 [9]. However, 20% of patients show discordance between *IL28B* genotype and the response, suggesting other factors including (TA)_n_ genotypes might be involved in HCV clearance. The lack of association between rs12979860 (*IL28B*) and ss469415590 (IFNL4) and the (TA)_n_ in this study may be unique to the Thai cohort as compared to other population. Another possibility may be that our study was under-powered and therefore could not identify such association.

The TA dinucleotide repeats, located precisely at the transcriptional start site of *IL-28B* gene, could be a biomarker for improved prediction of the response to interferon-based HCV treatment [10]. We demonstrated the correlation of (TA)_n_ genotypes with SVR. The (TA)_n_ ≥12 in the promoter region of *IL28B* was associated with HCV spontaneous clearance. It is not clear whether the variation originates from genetic or epigenetic mechanisms [19], and further studies will be needed to explore this observation in other populations. There have been several reports that implicated *IL28B* genotypes in inflammatory status and progression of fibrosis as measured by clinical parameters (ALT levels, alpha-fetoprotein, histological activity, levels of fibrosis and platelet-derived growth factor) [20, 21]. In our study, the baseline serum ALT level was significantly higher in patients with rs12979860 CC genotype compared to patients with non-CC genotype (*p = 0*.*011*). Similar observations were found in patients with ss469415590 TT/TT genotype compared to those with non-TT/TT genotype (*p = 0*.*028*). There were no significant differences in the baseline viral load between patients with rs12979860 CC and non-CC genotypes (*p = 0*.*075*), and patients with ss469415590 TT/TT genotype compared to those with non-TT/TT (*p = 0*.*083*). Finally, the rs12979860 and ss469415590 polymorphisms were not correlated with levels of fibrosis and platelet counts. To our knowledge, this study is the first to assess (TA)_n_ genotypes in relation to clinical characteristics in HCV-infected patients. Although (TA)_n_ genotypes were not associated with HCV viral load, liver inflammatory activity and liver fibrosis, they correlated with platelet counts (*p<0*.*001*). In clinical practice, genotyping HCV-infected patients to examine (TA)_n_ may predict the effectiveness of PEG-IFN/RBV therapy even before treatment has begun. Since approximately 2.2% of the Thai population has chronic HCV and financial burden can restriction access to needed antiviral treatment, the ability to reliably predict efficacy of therapy will be useful in the overall disease management.

Since many polymorphisms are associated with *IL-28B* and at least (TA)_n_ has been shown to regulate *IL-28B* transcription, this cytokine likely influence HCV clearance under PEG-IFN/RBV therapy and could also affect spontaneous clearance of acute HCV infection [15]. Administration of IL-28B has been shown to have antiviral effects [22–24], therefore lower expression of IL-28B as a result of unfavorable polymorphism might lead to a decrease in this effect.

Not only an individual’s genetic background plays an important role in the course of HCV infection, viral genotypes can also determine the course of infection. The observation that rs12979860 and ss469415590 polymorphisms were associated equally with the treatment outcome in response to PEG-IFN/RBV therapy in patients with HCV-1 infection, but not with HCV-3 and HCV-6, suggest that viral factors may also influence SVR in patients. Although significant differences between ethnicities in response to PEG-IFN/RBV therapy were reported [25], there were no significant associations between *IL28B* genotypes and response to PEG-IFN/RBV in patients infected with HCV genotype 2 or 3 [5]. In addition, some studies showed that the *IL28B* genotype did not predict response to treatment in HCV-5 and HCV-6 [26, 27].

In summary, our results demonstrated that (TA)_n_ genotypes was strongly linked to treatment response to PEG-IFN/RBV therapy in HCV-infected patients of Asian descent regardless of the viral genotype and led to a higher rate of SVR. Thus, prescreening for (TA)_n_ could assist clinical decision-making for the treatment of HCV infection and will be useful for making decisions on suitable regimens and treatment duration in patients in the forthcoming era of direct acting antiviral drugs.
